# SARS-CoV-2 Papain-like Protease Responsive ZnO/Daclatasvir-Loaded Chitosan/Gelatin Nanofibers as Smart Antimicrobial Medical Textiles: In Silico, In Vitro and Cell Studies

**DOI:** 10.3390/pharmaceutics15082074

**Published:** 2023-08-02

**Authors:** Mohamed Hamdi, Akram M. Elkashlan, Mohamed A. Hammad, Isra H. Ali

**Affiliations:** 1 Department of Pharmaceutics, Faculty of Pharmacy, University of Sadat City, Sadat City P.O. Box 32897, Egypt; mohamed.amira@fop.usc.edu.eg; 2 Department of Biochemistry, Faculty of Pharmacy, University of Sadat City, Sadat City P.O. Box 32897, Egypt; akram.elkashlan@fop.usc.edu.eg; 3 Department of Analytical Chemistry, Faculty of Pharmacy, University of Sadat City, Sadat City P.O. Box 32897, Egypt; m_abdelkhalek_eg@fop.usc.edu.eg

**Keywords:** microbial, viral, infection, medical textiles, nanofibers, daclatasvir, ZnO NPs, chitosan, gelatin

## Abstract

A significant number of deaths are reported annually worldwide due to microbial and viral infections. The development of protective medical textiles for patients and healthcare professionals has attracted many researchers’ attention. Therefore, this study aims to develop smart drug-eluting nanofibrous matrices to be used as a basic material for medical textile fabrication. First, chitosan/gelatin nanofibers were selected as the basic material owing to the wide antimicrobial activity of chitosan and the capability of gelatin to be hydrolyzed in the abundance of the papain-like protease (PLpro) enzyme secreted by SARS-CoV-2. Daclatasvir (DAC), an NS5A inhibitor, was selected as the model drug based on in silico studies where it showed high anti-SARS-CoV-2 potential compared to FDA-approved references. Due to their reported antimicrobial and antiviral activities, ZnO NPs were successfully prepared and incorporated with daclatasvir in chitosan/gelatin nanofibrous matrices through electrospinning. Afterward, an in vitro release study in a simulated buffer revealed the controlled release of DAC over 21 days from the nanofibers compared to only 6 h for free DAC. On the other hand, the abundance of PLpro induced the complete release of DAC from the nanofibers in only 4–8 h. Finally, the nanofibers demonstrated a wide antimicrobial activity against *S. aureus*, *E. coli*, and *C. albicans.*

## 1. Introduction

COVID-19, which was first detected in China in December 2019, has been vastly spreading worldwide. Furthermore, it has exhibited various mutations into more fierce infectious strains. Regrettably, WHO statistics updated to June 2023 have records of more than 750 million infected individuals, more than 6.5 million deaths, and more than 2500 new cases daily [1].

Each year, a huge number of deaths attributed to microbial infections are recorded worldwide. This is owed to the widespread abundance of drug-resistant microbial strains that trigger sepsis and consequently death. It is worth mentioning that more than 1/3 of all deaths reported worldwide are attributable to microbial infections and sepsis. Furthermore, patients with an impaired immune system, including diabetic and cancer patients, suffer from a high risk of microbial and viral infection complications, which in turn, can end up causing trauma and gangrene. Unfortunately, such complications may result in limb amputation or even death [2,3,4].

It is therefore highly recommended that preventive protocols are followed for microbial and viral infections since there are not clearly followed treatment protocols for various microbial infections, including for SARS-CoV-2. According to the WHO, approved preventive protocols include: (a) wearing face masks, (b) using protective medical clothes, e.g., head covers, gowns, overshoes, face shields, gloves, etc., and (c) social distancing, especially for healthcare professionals who are highly susceptible to viral and microbial infections. However, traditional medical textiles and face masks exhibit numerous drawbacks, e.g., headaches, migraines, and communication problems, owing to their poor porosity and consequent breathability that result in accumulating moisture and heat within the clothing’s microenvironment. Furthermore, they only serve as passive barriers lacking any bioactivity [5,6]. In addition, several face masks and medical textiles, e.g., N95, have been reported to fail in blocking some microbial and viral infections, e.g., SARS-CoV-2, although they have acted as efficient barriers for controlling the transmission of other microorganisms and respiratory viral strains possessing a size of more than 300 nm. On the other hand, some microorganisms, e.g., SARS-CoV-2, possessing a size range of 65–125 nm, were found to pass through such traditional medical textiles [7].

Although various techniques, e.g., etching and lithography, have successfully developed porous polymeric matrices that can act as medical textiles possessing controlled pore shapes and sizes, they failed to fabricate cheap textiles on a large scale owing to their dependence on specialized equipment and advanced environments, e.g., clean room facilities [7].

In contrast, electrospun polymer nanofibers possess numerous advantages, such as: (a) being fabricated in an eco-friendly and cost-effective way, (b) being prepared in a facile way on a large scale, and (c) possessing a controllable pore size and volume, which enhances high breathability, ventilation, and adequate airflow. Therefore, nanofibers can be easily tailored to act as medical textiles that can control the wide spread of microorganisms, particularly minute viral strains, including SARS-CoV-2 [8,9,10,11]. Furthermore, nanofibers are characterized as being able to preserve their porous structure as well as their structural integrity even after being exposed to a humidity-saturated environment, unlike traditional face masks [6].

Chitosan (CS) is a positively charged, chemically modified derivative of chitin. It is the second-most prevalent polysaccharide and biopolymer found globally after cellulose. CS can be prepared through chitin deacetylation through either the alkaline or enzymatic hydrolysis route. Chitin is converted into chitosan to increase its solubility and consequently its facile usage in the fabrication of biomaterials. CS has been widely used as a promising biomaterial in various biomedical applications owing to its diverse advantageous features, e.g., (a) being biocompatible and biodegradable, (b) exhibiting wide-spectrum antiviral and antimicrobial activities, (c) enhancing tissue restoration and stimulating cell adhesion as well as proliferation, (d) possessing hemostatic activity, (e) demonstrating minimal cytotoxicity and immunogenicity, and (f) having no carcinogenicity [9,12].

Gelatin (GL) is another well-known and widely utilized FDA-approved naturally derived biopolymer. It is a hydrophilic biopolymer that possesses negatively charged functionalities within its protein structure. It is characterized by being (a) highly biodegradable and biocompatible, (b) exhibiting a low level of antigenicity, irritability, and immunogenicity, and (c) demonstrating no carcinogenicity nor toxicity. GL is derived from collagen, another natural protein, through either alkaline or acidic hydrolysis. GL can be easily cross-linked to other biopolymer molecules or be chemically modified, with its traits attributed to the abundance of various functional moieties within its structure. In addition, GL can be used in the designing and developing of smart biomaterials that would respond to protease enzymes, including papain. Hence, it can be incorporated within nanocarrier structures to control the release of drugs as a response to the abundance of SARS-CoV-2 infection [13,14].

Earlier studies involving CS and GL combinations proved the high efficiency of such a composite matrix as a biomaterial that can be utilized in various biomedical applications. This was attributed to the electrostatic interactions detected between the CS positively charged groups and the GL negatively charged moieties [15,16].

Daclatasvir (DAC) is an FDA-approved antiviral medication that has proven its high efficiency in hepatitis C (HCV) treatment through the inhibition of the NS5A protein [17]. In addition, several initial in silico studies were performed to investigate the activity of various NS5A inhibitors towards inhibiting SARS-CoV-2 enzymes. In addition, initial in silico and in vivo studies were conducted to examine the effectiveness of various NS5A inhibitors, including DAC, in inhibiting SARS-CoV-2 proteases, particularly papain-like protease (PLpro). Their findings indicated a strong potential for DAC to function as an antiviral agent against SARS-CoV-2 by inhibiting PLpro, one of the most important and invasive SARS-CoV-2 proteases. These preliminary results suggest the high potential of in-detail evaluations of the potential of DAC to act as a promising antiviral medication against SARS-CoV-2 through the inhibition of PLpro [18,19,20,21].

Through previous studies, zinc oxide nanoparticles (ZnO NPs) proved their efficiency as antiviral agents against different viral infections, e.g., herpes simplex virus type III and H1N1 influenza virus [22,23]. Although the most commonly used metal and metal oxide-based nanoparticles, e.g., gold and silver, lack biocompatibility, ZnO nanoparticles have demonstrated their high biocompatibility alongside their high stability and cost-effectiveness [24]. Furthermore, ZnO NPs are characterized by possessing a wide spectrum of antimicrobial activities in addition being inert towards pharmaceuticals. Hence, they are considered promising materials to be used in combination with other antiviral medications. In addition, previous in silico and in vitro studies have demonstrated the promising anti-SARS-CoV-2 activity of ZnO NPs [25].

**Scheme 1:**
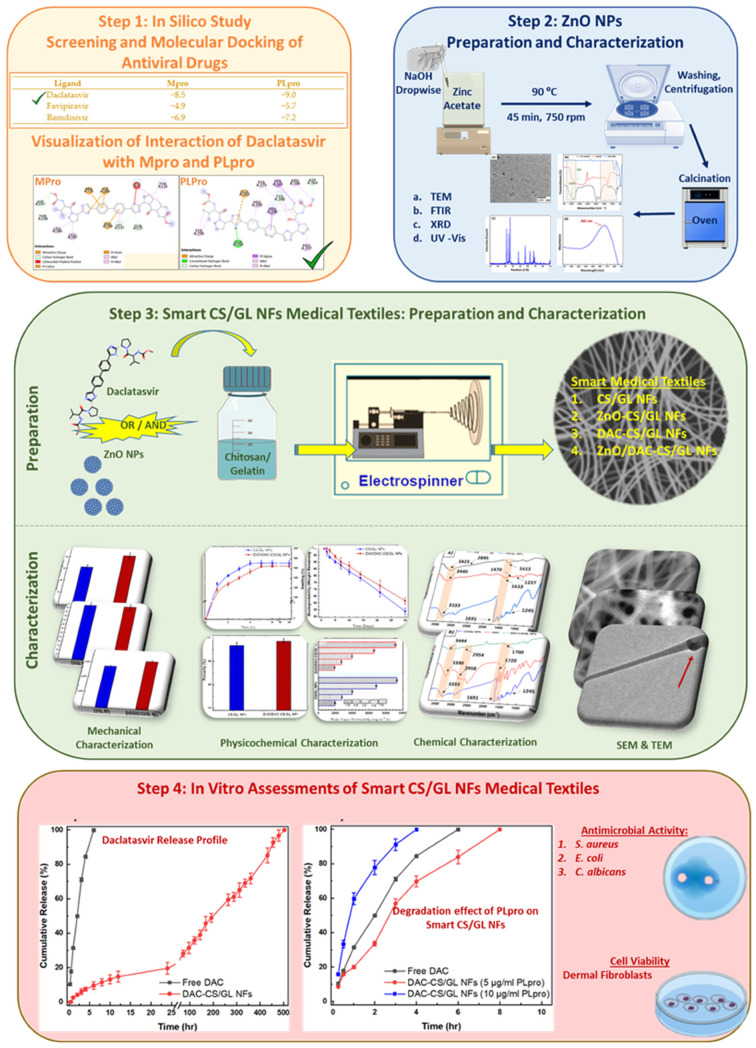
A summary of the main stages of the workflow of the study.

Hence, this study aimed to compare the in silico inhibitory activity of daclatasvir (DAC) towards PLpro to remdesivir and favipiravir; two approved antiviral drugs for SARS-CoV-2 treatment. ZnO NPs were also optimized and prepared through the sol-gel technique. Afterward, ZnO NPs and DAC were both incorporated inside CS/GL nanofibrous matrices (ZnO/DAC-CS/GL NFs) through electrospinning, in which the CS/GL NFs acted as smart medical textiles that can be biodegraded to release the incorporated antiviral agents as a response to the abundance of SARS-CoV-2 PLpro. Finally, the developed nanofibers were fully characterized and assessed for their antimicrobial activity as well as their cell viability using human fibroblasts. The workflow of the study has been summarized in Scheme 1.

## 2. Materials and Methods

### 2.1. Materials

Low molecular weight chitosan, type A gelatin, zinc acetate, and sodium hydroxide were all obtained from Acros, Geel, Belgium. Daclatasvir hydrochloride, glacial acetic acid (99.7%), absolute ethanol (99%), saline phosphate-buffered saline (PBS, pH 5.5), PLpro enzyme, and cellulose dialysis membrane (cut-off 12,000 MW) were purchased from Sigma-Aldrich, Taufkirchen, Germany.

Difco LB broth agar and medium were purchased from the Beckman Dickinson Company, Franklin Lakes, NJ, USA. *Staphylococcus aureus* (ATCC 6538) as a Gm +ve bacteria, *Escherichia coli* (ATCC 8739) as a Gm −ve bacteria, and *Candida albicans* (ATCC 10231) were selected to investigate the antimicrobial activity of the prepared nanoformulations. Finally, cell viability was estimated using dermal fibroblasts, where a 3-(4,5-dimethylthiazol-2yl)-2,5-diphenyl tetrazolium bromide (MTT) assay kit was obtained from Sigma-Aldrich, Wuxi, China.

### 2.2. In Silico Comparison between Daclatasvir and the Reference Drugs

#### 2.2.1. Ligand Files Preparation

Daclatasvir, along with two reference drugs, namely favipiravir and remdesivir, were downloaded as sdf files in their minimized energy form from the Pubchem database (https://pubchem.ncbi.nlm.nih.gov/ (accessed on 7 December 2022)). This was carried out to predict the efficiency of the selected drug as an anti-SARS-CoV-2 agent compared to the already approved reference drugs.

#### 2.2.2. Preparation of the Mpro and PLpro Enzymes

Main protease (Ppro) and papain-like protease (PLpro) were investigated and then downloaded from the Swiss model (https://swissmodel.expasy.org/ (accessed on 8 December 2022)) [26,27] and protein database bank (https://www.rcsb.org/ (accessed on 7 December 2022)) as pdb files, respectively. Then, the obtained enzymes files were refined where the co-crystal ligands were removed. Finally, the enzyme-only files were downloaded as pdb files using PyMol software version 2.2.3.

#### 2.2.3. Molecular Docking and Visualization

Molecular docking of the three tested drugs into each of Mpro and PLpro as receptors was performed through the CB-Dock docking server (http://clab.labshare.cn/cb-dock/php/index.php (accessed on 9 December 2022)) [28,29]. Afterward, drug–enzyme interaction files for each tested drug was downloaded separately as a mol2 file according to the used server recommendations. Finally, Discovery Studio-19 software version 19.1.0.18287 was used to visualize the interaction between each enzyme and each drug separately. The studied interactions involved aromatic ring centers, hydrophobic interactions, H-bonding, covalent bonds, parallel and perpendicular p-stacking, etc. The enzyme–drug binding interaction was evaluated based on the calculated binding free energy (DG).

### 2.3. Preparation and Characterization of the ZnO Nanoparticles

#### 2.3.1. Preparation of the ZnO NPs

ZnO NPs were prepared using the sol-gel technique according to the previously reported protocols with minor adjustments [25,30]. Briefly, the chemical reaction was performed by adding 1M of sodium hydroxide aqueous solution slowly and dropwise into 100 mL of 300 mM of zinc acetate aqueous solution until the pH of the reaction was adjusted to 9.5. Then, the reaction was left on a hot plate magnetic stirrer adjusted at 750 rpm for 45 min at 90 °C to form a ZnO nanosuspension. Afterward, ZnO NPs were centrifuged at 12,000 rpm at 4 °C for 15 min. Then, ZnO nanoparticles were retrieved, washed with distilled water (3×) followed by absolute ethanol (3×), and then centrifuged again for 15 min at 4 °C and 12,000 rpm. Finally, the nanoparticles were obtained, dried, and then calcinated in an electric oven at 250 °C for 6 h.

#### 2.3.2. Characterization of the ZnO NPs

The obtained ZnO NPs were characterized morphologically, chemically, and physically.

For morphological investigation, the ZnO NP sample was examined using transmission electron microscopy (TEM) (JEOL JEM-1230, Tokyo, Japan), whereby a drop of the diluted nanosuspension in deionized water was placed on a carbon-coated copper mesh grid, then left to dry for 10 min, stained with phosphotungstic acid, and finally examined [25].

For chemical characterization, FTIR spectroscopy (Thermo Scientific, Waltham, MA, USA) was utilized to investigate the chemical structure of the developed ZnO NPs within the spectrum range of 400–4000 cm^−1^ [31].

For the determination of the ZnO NPs crystallite size and shape, XRD (Philips X’Pert system, Amsterdam, The Netherlands) was used. XRD analysis was performed using Cu Kα radiation at 30 mA and 40 kV with a 2θ value covering the range of 10–80°. Afterward, the ZnO NPs crystallite size was then estimated using Scherer’s equation displayed in Equation (1), where d is the crystallite size of the ZnO NPs, K represents the dimensionless shape factor (0.9), 𝜆 represents the wavelength estimated to be 0.154060 nm, β represents the peak line broadening at the midway of the maximum intensity (FWHM), and θ represents Bragg’s angle of diffraction [32].

(1) $$d=\frac{K\lambda }{\beta Cos\;\theta }$$

The diluted ZnO NPs nanosuspension in deionized water was scanned in the range of 200–400 nm using a UV–Visible double beam spectrophotometer (Evolution 201, Thermo Scientific, Horsham, UK), where deionized water was used as the blank. Afterward, the band gap of the developed ZnO NPs was calculated using Max Planck’s equation displayed in Equation (2), where h represents the Planck constant (4.14 × 10^−15^ eV s), c is the speed of light (2.99 × 10^8^ ms^−1^), and 𝜆 is the maximum detected wavelength [25,33].

(2) $$E_{g}=\frac{hc}{\lambda }$$

Finally, the zeta potential of the developed ZnO NPs was estimated using the Nanosizer ZS Series (Malvern Instruments, Malvern, UK) as an indication for the nanoparticles’ surface charges to investigate their stability in nanosuspension. The nanosuspension was diluted 10-fold before being measured through the dynamic light scattering technique (DLS) [25].

### 2.4. Preparation of the Unloaded and Loaded CS/GL NFs

#### 2.4.1. Preparation of the Electrospinning Solutions

Chitosan/Gelatin NFs were developed through electrospinning according to previously tested protocols with slight adjustments [34]. Briefly, both chitosan and gelatin were dissolved in 1% acetic acid aqueous solutions to prepare CS (3% *w*/*v*) and GL (25% *w*/*v*) solutions, respectively. The CS solution was obtained at room temperature, while the GL solution was obtained at 40 °C, respectively. After the complete dissolution of both solutions, the CS and GL solutions were mixed in a ratio of 1: 1 and left on a magnetic stirrer at room temperature for 2 h to obtain a homogenous solution.

To prepare the loaded CS/GL solutions, either DAC or the ZnO NPs were added separately to the CS/GL solution and then stirred to obtain their corresponding DAC-CS/GL solution and ZnO-CS/GL suspension, respectively. Each added amount was equivalent to 10% *w*/*w* relative to the total polymer weight in the CS/GL solution. Finally, ZnO/DAC-CS/GL solution was prepared by adding both ZnO and DAC into the CS/GL solution using the same previously mentioned amounts.

#### 2.4.2. Preparation of the Unloaded and Loaded Electrospun Nanofibers

Each solution was fed separately into a 5 mL syringe and then electrospun into their corresponding nanofibrous matrices using NANON–O1A (Mecc, Fukuoka, Japan), according to the previously mentioned method [2,9,10,35]. The optimum parameters were 1 mL/h feed rate, 19 KeV applied voltage, 15 cm distance between syringe and collector, room temperature, and 35% humidity. The produced nanofibrous matrices were CS/GL NFs, DAC-CS/GL NFs, ZnO-CS/GL NFs, and ZnO/DAC-CS/GL NFs, respectively.

#### 2.4.3. Crosslinking of the Unloaded and Loaded Electrospun Nanofibers

The different fabricated nanofibrous matrices were chemically cross-linked according to the previously reported protocol with slight modifications to increase their durability and water stability [34]. Briefly, the nanofibers were placed on a mesh to be exposed to vapors of 25% glutaraldehyde aqueous solution. The nanofibers were left for 6 h for crosslinking and then placed in distilled water for 15 min to test for their stability in water. Finally, they were frozen and then freeze-dried to obtain any remnant liquids.

### 2.5. Characterization of the Unloaded and Loaded CS/GL NFs

#### 2.5.1. Morphological Characterization

In order to investigate the external and internal morphological features of the developed nanofibers, SEM (Nova Nano SEM, FEI, Rock Hill, SC, USA) and TEM (JEM-2100F; JEOL, Peabody, MA, USA) were used, respectively. SEM images were analyzed to estimate the diameter and polydispersity index (PDI) of both the unloaded and loaded nanofibers through ImageJ software version 1.52v. Furthermore, SEM imaging was used to investigate the morphological features of these nanofibers after being cross-linked. On the other hand, TEM imaging was used to detect the incorporation of the ZnO NPs within the nanofibrous matrices [36].

#### 2.5.2. Chemical Characterization 

Fourier transform infrared spectroscopy (FTIR) was used to investigate the chemical composition of both unloaded CS/GL NFs in order to study the interaction between both the CS and GL structures. Furthermore, the loaded CS/GL NFs were investigated to prove the successful loading of DAC within the nanofibrous matrices. This study was carried out using FTIR spectroscopy (Thermo Scientific, Waltham, MA, USA) within the spectrum range of 400–4000 cm^−1^ [34].

#### 2.5.3. Physicochemical Characterization

The physicochemical properties of the fabricated nanofibrous matrices were studied in terms of their biodegradability, swellability, water vapor permeability (WVP), and porosity. Biodegradability was evaluated through immersing pre-weighed samples of the different nanofibrous matrices in a pH 5.5 PBS solution at 37 °C inside a shaking incubator. Afterward, the tested samples were removed, dried, and then weighed again. This was repeated daily until the end of the experiment. Then, Equation (3) was used to calculate each sample’s remaining weight percentage, where Wi represents the initial weight of each sample at the beginning of the experiment, while Wf is the final weight of each sample estimated every day [37]. Finally, a plot was constructed representing the percentage of weight remaining versus the corresponding time points.

(3) $$Remaining\;Weight\;\%=\left(\frac{Wf}{Wi}\right)\times 100$$

To calculate the swellability of the different fabricated nanofibrous matrices, samples of each type of nanofiber were weighed at the beginning of the experiment (Wd) before being placed in a buffer (pH 5.5 PBS) at 37 °C inside a shaking incubator for the whole experiment duration. Following this step, at specific time points, the samples were taken out of the setup and placed on a dry filter paper to remove any excess PBS before being weighed again (WS). These steps were repeated until no further weight changes were detected. Finally, the swelling percentage of each nanofiber was assessed using Equation (4) and then plotted against their time points [37].

(4) $$Swelling\;\%=\left(\frac{Ws-Wd}{Wd}\right)\times 100$$

Water vapor permeability (WVP) or breathability is an important aspect that can be used to estimate the capability of the tested nanofibrous matrices in permitting the passage of water vapor. WVP can be determined using the ASTM E96 desiccant protocol [38,39]. Briefly, the experimental setup included placing a weighed amount of dry silica gel into an Erlenmeyer conical flask, which was then covered with a piece of one of the tested nanofibrous matrices. Afterward, the flasks were kept in a 75% humidity-saturated environment during the experiment duration. The flasks were taken and the weight change was estimated at specific time intervals. Finally, the WVP of each of the nanofiber types was determined using Equation (5) before being plotted against their corresponding time points, where ∆W represents the weight change of silica indicating the WVP through the samples, A is the surface area of the sample, and ∆t represents the time change during the experiment.

(5) $$WVP=\left(\frac{∆\;W}{A}\right)\times ∆t$$

A pycnometer (Ultrapyc 1200 e, Quantachrome Instruments, Boynton Beach, FL, USA) was used to estimate the porosity per volume for each of the tested nanofibrous matrices. Pieces of each nanofiber type of a known size were weighed and then placed in the container of the instrument [40]. Then, the porosity per volume, as well as the porosity %, were calculated using Equations (6) and (7), respectively, where VF represents the volume of each of the assessed samples detected using helium gas through the pycnometer.

(6) $$Porosity\;per\;Volume=1-\frac{VF}{Total\;Volume(Length\times Width\times Thickness)}$$

(7) Porosity %=Porosity per Volume×100

#### 2.5.4. Mechanical Characterization

The different nanofibrous matrices were assessed for their mechanical properties in terms of their ultimate tensile strength, extensibility percentage, and Young’s modulus using a universal testing machine (Epsilon, Irving, TX, USA), as reported previously [35]. Samples of 60 mm × 2 mm × 0.5 mm representing the length, width, and thickness, respectively, were used.

### 2.6. In Vitro Daclatasvir Release Profile from the CS/GL NFs

The daclatasvir release profile from the developed CS/GL NFs was investigated using the dialysis bag method, where phosphate buffer (pH 5.5) was used as the receptor medium to maintain sink conditions. Briefly, a predetermined weight of the loaded nanofibers was kept in a dialysis bag and then inserted into a falcon tube containing 20 mL of the used buffer. Afterward, the experimental setup was then kept at 37 °C inside a shaking water bath to monitor the daclatasvir release profile throughout the whole experiment duration. Then, at specific time points, samples of the receptor medium were withdrawn to be analyzed using a UV–Visible spectrophotometer (Evolution 201, Thermo Scientific, Horsham, UK) at a wavelength of 234 nm to calculate the drug concentration using Equation (8), where Cn is the expected concentration of the n^th^ sample, while Cn means represents the estimated concentration, respectively. A is the withdrawn sample’s volume, V is the dissolution medium volume, and n−1 is the total volume. Finally, Cs demonstrates the combined concentrations of all the previously withdrawn samples proceeding with the current sample. The daclatasvir release pattern from the CS/GL NFs was then compared to that of free daclatasvir. The release profile was repeated three times, following which a plot of the cumulative release percentage versus time was constructed.

(8) $$Cn=Cn\;means+A/V\;\sum_{s=1}^{n-1}Cs\;means$$

Finally, various models, such as the zero-order, first-order, Higuchi, Korsmeyer–Peppas, Hixson–Crowell, Hopfenberg, and Baker–Lonsdale models were all followed to assess the release kinetics of daclatasvir.

### 2.7. In Vitro PLpro Activity towards Smart CS/GL NFs

The in vitro activity of papain-like protease (PLpro) towards the fabricated smart CS/GL NFs was studied, where varying concentrations of PLpro were added into pre-weighed samples of DAC-CS/GL NFs before being incubated at different time points at 37 °C. Then, the supernatant was collected to estimate the released amount of DAC using a UV–Visible spectrophotometer (Evolution 201, Thermo Scientific, Horsham, UK) at a wavelength of 234 nm. The cumulative amount of the released drug was plotted versus time to study the influence of PLpro in breaking down the fabricated smart nanofibers.

### 2.8. Antimicrobial Activity of Unloaded and Loaded CS/GL NFs

This study assessed the antimicrobial activities of free DAC, ZnO NPs, ZnO/DAC combination, CS/GL NFs, DAC-CS/GL NFs, ZnO-CS/GL NFs, and ZnO/DAC-CS/GL NFs against *Staphylococcus aureus* as a Gram-positive bacteria, *Escherichia coli* as a Gram-negative bacteria, and *Candida albicans* as a fungus, respectively. This study was conducted using the diffusion agar technique, where 6 mm holes were made within the agar plates in which 100 µL of each sample was added separately, each in a separate hole. Afterward, the inhibition zone was measured to evaluate the antimicrobial activity of each tested sample against each of the examined pathogens.

### 2.9. In Vitro Cell Viability and Biocompatibility of the ZnO/DAC-CS/GL NFs

Both CS/GL NFs and ZnO/DAC-CS/GL NFs were assessed for their biocompatibility through assessing fibroblast cell viability using the MTT assay, as previously described in ISO 10993-5 protocol [9,36]. This experiment involved using a dermal fibroblast cell line (ATCC CRL-2522), whereby 200 μL of the cell suspension (1 × 10^5^ cells) was plated for each type of nanofiber matrix per well in a 24-well plate before being incubated for 24 h. Afterward, the medium was withdrawn and the wells were washed with MEM (*w*/*o*) FCS. Then, MTT reagent (200 μL) was added to each well and then incubated for 6 h inside a 5% CO_2_ incubator. Afterward, DMSO was added to each well and mixed well before being left for 60 s to measure the optical density of the viable fibroblasts at a λ of 595 nm using a spectrophotometer, where DMSO was used as the blank. The purple color development of the formazan crystals indicated the presence of viable cells. Finally, the cell viability percentage was determined using Equation (9).

(9) $$Cell\;Viability\%=\left(\frac{Mean\;Optical\;Density}{Control\;Optical\;Density}\right)\times 100$$

### 2.10. Statistical Analysis

The experiments were run in triplicate, where all results were presented as the mean ± standard deviation. One-way analysis of variance (ANOVA) for multigroup experiments and Student’s *t*-test for two-group experiments were performed to detect any significant differences within the data. Calculations were carried out using GraphPad Prism software version 6.

## 3. Results

### 3.1. In Silico Studies of the Anti-SARS-CoV-2 Activity of Daclatasvir

#### 3.1.1. Molecular Docking of Different Ligands with Mpro and PLpro

The CB-Dock docking server was used to calculate the Gibbs free energy (http://clab.labshare.cn/cb-dock/php/index.php (accessed on 9 December 2022)) [28,29] to investigate the strength of the interactions between either the Mpro or PLpro receptors on one side and its different ligands, which include daclatasvir, favipiravir, and remdesivir, on the other side, as shown in Table 1. Favipiravir and remdesivir were selected to be references for daclatasvir owing to their reported FDA-approved anti-SARS-CoV-2 activity. In this study, it was observed that daclatasvir possessed the highest Gibbs free energy with both Mpro and PLpro when compared to both favipiravir and remdesivir, which concluded that daclatasvir exhibits the highest inhibitory activity against both enzymes. This proves that daclatasvir would be a promising candidate as an anti-SARS-CoV-2 agent. These results are in good agreement with what has been reported previously, where various in vitro and in silico studies suggested the promising anti-SARS-CoV-2 activity of NS5A inhibitors through a few drug repurposing studies [18,19,21]. Hence, DAC is considered a promising candidate for this study. Furthermore, it was observed that the Gibbs free energy of daclatasvir with PLpro was higher than Mpro, which suggests that most of the anti-SARS-CoV-2 activity of DAC owes to its inhibitory activity towards PLpro compared to Mpro. Hence, PLpro was selected for further in vitro studies.

#### 3.1.2. Visualization of the Interaction between Daclatasvir with Either Mpro or PLpro

**Figure 1:**
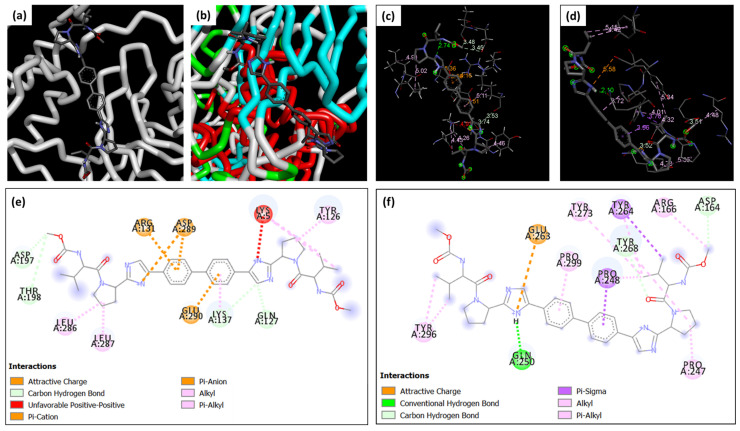
Overview of the interaction of DAC with (**a**) Mpro and (**b**) PLpro within the active site of each enzyme. Three-dimensional visualization of the interaction of DAC with (**c**) Mpro and (**d**) PLpro, and a 2D diagram for the bonds and interactions detected between DAC and (**e**) Mpro and (**f**) PLpro.

Discovery Studio-19 software version 19.1.0.18287 was used to investigate and visualize the interactions detected between either Mpro or PLpro with daclatasvir, as shown in Table 2 and Figure 1. Table 2, as well as Figure 1a,b display the detected interactions of daclatasvir with both Mpro and PLpro, respectively, emphasizing the involved amino acids in each enzyme, as well as the interactions or bonds along with their calculated distance. In this study, it has been inferred that daclatasvir was able to interact with chain A of each Mpro and PLpro through eleven amino acids. However, it was inferred from the results that interactions detected between DAC with PLpro are stronger than that detected between DAC and Mpro. These results match the results of the Gibbs free energy displayed in Table 1 that was outlined in Section 3.1.1.

For instance, DAC and PLpro interactions involve a hydrogen bond which is considered a strong bond through GLN (A: 250), as shown in Table 2 and Figure 1d,f. This was considered a strong interaction owing to the high difference in electronegativity between the hydrogen atom and the more electronegative atom. However, this type of interaction has not been detected with Mpro, as shown in Table 2 and Figure 1c,e.

In addition, DAC interacts with PLpro through two π–sigma bonds via PRO (A: 248) and TYR (A: 264), as demonstrated in Table 2 and Figure 1d,f. This type of bond is formed between a sigma bond and a π–bond, so it is considered as a strong type of interaction owing to the overlap of the electron clouds of both types of bonds together. On the other hand, no π–sigma bonds were detected between DAC and Mpro, as illustrated in Table 2 and Figure 1c,e.

Furthermore, the number of π–alkyl bonds detected between DAC and PLpro was found to be higher than the number of alkyl–alkyl bonds, unlike Mpro, which displayed a greater quantity of alkyl–alkyl bonds. π–alkyl interactions are relatively strong compared to alkyl–alkyl interactions. This is attributed to the good interaction between the electron-rich π–system of the aromatic ring from one side and the alkyl group on the other side. However, alkyl–alkyl interactions are typically weaker, owing to the relatively low polarity of the involved alkyl groups.

Hence, these results confirm that the mechanisms underlying the anti-SARS-CoV-2 activity of DAC would be more prevalent through the inhibition of PLpro rather than Mpro.

### 3.2. Preparation and Characterization of the ZnO NPs

**Figure 2:**
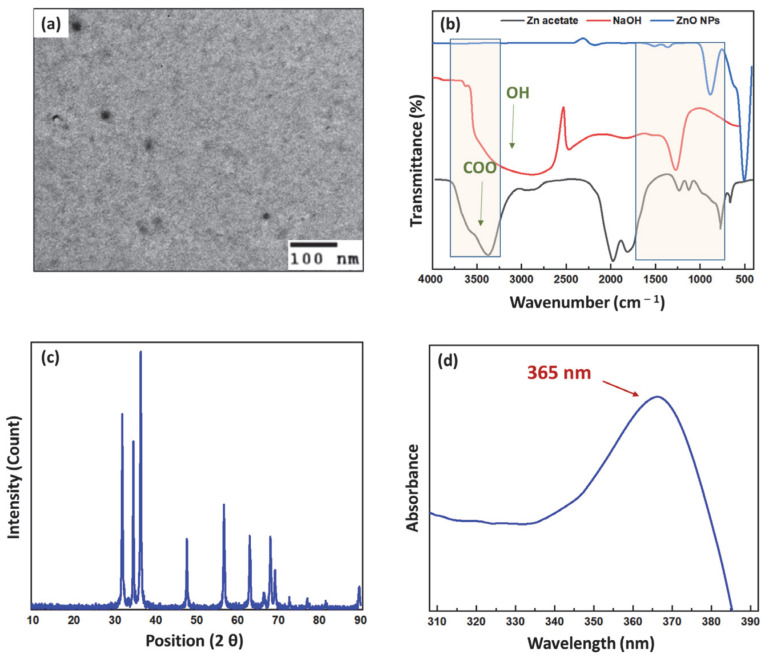
(**a**) TEM image showing the hexagonal, semi-spherical shape of ZnO NPs. (**b**) FTIR spectra for Zn acetate, NaOH, and ZnO NPs illustrating the successful formation of ZnO NPs. (**c**) XRD diffractogram showing the crystallite size and shape of the ZnO NPs. (**d**) UV spectrum of ZnO NPs showing the wavelength of maximum absorbance.

ZnO NPs have been prepared successfully using wet chemical methods, as illustrated previously [25]. This has been confirmed through the full characterization of the developed nanoparticles. For instance, the TEM imaging displayed in Figure 2a confirmed the successful formation of hexagonal or semi-spherical ZnO NPs possessing sizes of around 15–20 nm.

Furthermore, Figure 2b demonstrates the FTIR spectra for zinc acetate, the precursor used, sodium hydroxide, and the ZnO NPs. The zinc acetate spectrum demonstrated a characteristic peak in the range of 3000–3500 cm^−1^, which was attributed to the carboxylic group. On the other hand, the sodium hydroxide spectrum also showed a broad band in the range of 2500–3000 cm^−1^ owing to the hydroxyl group. By comparing their spectra to that of the ZnO NPs, it can be observed that both peaks have disappeared in the ZnO NPs spectrum, which confirms the successful completion of the chemical reaction between zinc acetate and sodium hydroxide, resulting in the formation of the ZnO NPs. Furthermore, the disappearance of any peaks in the range of 2500–3500 cm^−1^ was also deemed to confirm the proper washing, purifying, and calcination of the developed ZnO NPs. In addition, the intense peak appearing in the fingerprint region at 400 cm^−1^ in the ZnO NPs corresponding to Zn–O stretching confirms the successful synthesis of these ZnO NPs. These results are found to be in a good match with previously reported spectra in the literature [25,33,41].

The X-ray diffractogram displayed in Figure 2c shows the characteristic peaks of the hexagonal-shaped crystalline ZnO NPs at 2θ values of 31.75, 34.45, 36.25, 47.55, 56.58, 62.89, 66.32, 67.94, and 69.05, respectively. The crystallite size was determined using Scherrer’s equation to be approximately 17.38 nm. This was found to be highly consistent with the size results obtained from TEM imaging. Furthermore, these findings are in good accordance with previously reported results [25,33].

Figure 2d demonstrates the UV–Vis spectrum of the diluted nanosuspension of ZnO NPs in deionized water. It was inferred that the excitonic peak appearing at the wavelength of 365 nm indicated that ZnO had been synthesized at the nanoscale range compared to that of the bulk observed at 380 nm. Furthermore, the band gap calculated using Max Planck’s equation revealed a value of 3.28 eV, confirming the formation of ZnO in the form of nanoparticles. These findings were found to be in good agreement with the previously reported literature [25,33].

Finally, zeta potential measurements that were carried out using the DLS technique demonstrated that the developed ZnO NPs possess a positively charged surface of 53.42 ± 3.88 mV value. The high positive charge can be attributed to the successful retrieval of ZnO NPs in a highly purified form with no hydroxyl groups after the removal of any excess sodium hydroxide, as well as proper calcination to remove excess water molecules. Additionally, a high surface charge indicates the high stability of the produced ZnO NPs, thereby preventing their aggregation and cake formation. These results were found to be in good agreement with previously reported results [9,10,25].

### 3.3. Preparation and Characterization of the Unloaded and Loaded CS/GL NFs

Both unloaded and loaded CS/GL NFs have been successfully developed through the electrospinning process into uniform matrices using their corresponding prepared solutions as shown in Table 3, where their composition and solution parameters are demonstrated. Then, they have been successfully cross-linked using glutaraldehyde vapors. This was confirmed after soaking them in distilled water for 15 min, through which they proved their high stability and durability before being further characterized and assessed.

#### 3.3.1. Morphological Characterization

**Figure 3:**
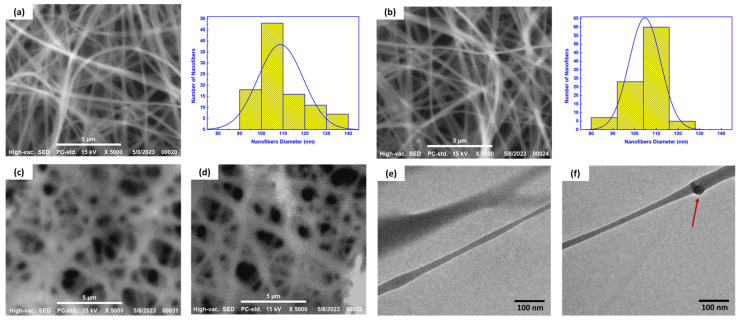
(**a**,**b**) SEM imaging and histograms for nanofiber analysis of the CS/GL NFs and ZnO/DAC-CS/GL NFs, respectively, (**c**,**d**) SEM imaging of the cross-linked nanofiber matrices of the CS/GL NFs and ZnO/DAC-CS/GL NFs, respectively, (**e**) TEM imaging of DAC-CS/GL NFs, and (**f**) TEM imaging of ZnO-CS/GL NFs showing the incorporated ZnO NPs within the nanofiber matrices. Red arrow pointing at ZnO NPs within nanofibrous matrices.

Both CS/GL NFs and ZnO/DAC-CS/GL NFs have been investigated morphologically using SEM imaging to examine the nanofibers’ uniformity, diameter, and PDI, as shown in Figure 3a,b, respectively. Based on the SEM images obtained, it was inferred that both unloaded and loaded nanofibrous matrices possess uniform non-beaded nanofibers. This confirmed that the incorporation of both DAC and ZnO NPs was accomplished successfully without negatively affecting the uniformity of the produced nanofibers. Furthermore, histograms based on ImageJ software analysis revealed that both nanofibrous matrices possess a narrow dispersity index with a uniform bell shape nanofiber distribution. However, it was also observed that incorporating ZnO NPs and DAC led to an observed decrease in nanofiber diameter from 111 ± 9.5 nm with a size distribution of 99–132 nm to 105 ± 7 nm with a size distribution of 82–117 nm, as shown in Figure 3a,b, respectively. This can be attributed to the decrease in the amount of polymer amount ejecting from the spinneret during the spinning of the loaded CS/GL solution compared to that observed of the unloaded one, resulting in the reduction of the ejected nanofibers diameter. This was found to be in good agreement with what has been reported in previous studies [9,10,34].

In addition, it was found that the incorporation of both ZnO NPs and DAC had an observed influence in decreasing the polydispersity index from 0.803 for the CS/GL NFs compared to 0.511 for the ZnO-DAC/CS/GL NFs, respectively.

SEM imaging was then performed again for both the CS/GL NFs and the ZnO/DAC-CS/GL NFs to investigate their morphological features after being cross-linked and soaked in distilled water, as shown in Figure 3c,d, respectively. From this analysis, it was found both nanofibers were successfully cross-linked. Furthermore, they were proven to preserve their structural integrity as uniform and robust nanofibrous porous matrices even after being soaked in an aqueous medium. Altogether, this proves that these developed nanofibrous matrices can be easily used in further assessments and applications.

Finally, TEM imaging was used to investigate the internal morphology of both the DAC-CS/GL NFs and ZnO-CS/GL NFs. It has been found that DAC was completely dissolved in the CS/GL electrospinning solution homogeneously, as no aggregations nor drug precipitation was detected, as shown in Figure 3e. On the other hand, ZnO NPs were found to be incorporated successfully within the CS/GL NF matrices without disrupting their uniformity, as shown in Figure 3f.

#### 3.3.2. Chemical Characterization

**Figure 4:**
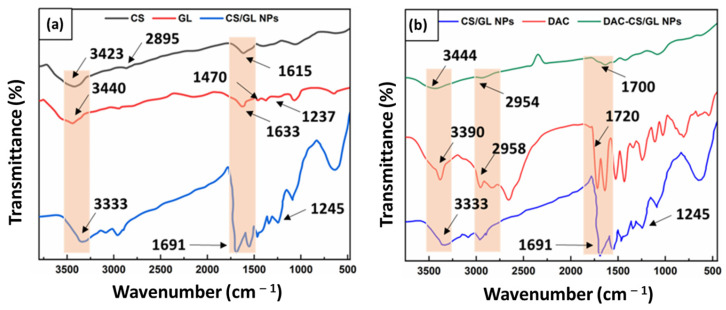
FTIR spectra of: (**a**) CS/GL NFs showing a successful complexation between chitosan and gelatin within the nanofiber matrices, and (**b**) DAC-CS/GL NFs showing a successful incorporation of DAC within the nanofiber matrices.

Figure 4a,b display the FTIR spectra of the CS/GL NFs and DAC-CS/GL NFs, respectively. In Figure 4a, the chitosan spectrum exhibited a broad peak at 3423 cm^−1^, which was determined to correspond to the stretching of both the O–H and NH_2_ groups. Both the amide I band, which reflects the abundance of C=O within the NHCOCH_3_ group, and the amide II band, which confirms C–O stretching along with N–H bending, were identified through the abundance of a distinctive peak at 1615 cm^−1^. Multiple peaks observed at 2895 cm^−1^ represent the presence of both the symmetric CH_3_ as well as asymmetric CH_2_ stretching [34,42,43,44].

As shown in Figure 4a, GL possesses three observable peaks at 1633 cm^−1^, 1470 cm^−1^, and 1237 cm^−1^, which correspond to C=O group stretching, N−H group bending, and C−N group stretching, respectively. Furthermore, a wide peak was observed in the 3400-3500 cm^−1^ range, which was attributed to both the NH_2_ and OH groups stretching within the GL chemical structure. The obtained GL spectrum was found to be in accordance with what has been previously reported in the literature [44].

Interestingly, the FTIR spectrum of the CS/GL NFs demonstrated in Figure 4a revealed that the bands of both the C=O and NH_2_ groups have been shifted to 1691 cm^−1^ and 3333 cm^−1^, respectively. This provides evidence for the formation of bonds among the CS and GL structures, confirming the capability of the OH, NH_2_, and C=O groups within the GL structure in forming bonds with the OH and NH_2_ groups in the CS structure. This was further justified by the polar structures of both CS and GL, which allow for the formation of charges on their functional groups upon their solubilization, resulting in the formation of polar bonds. This returns to the cationic structure of CS and the anionic structure of GL, respectively. These findings are supported by previous studies in the literature [34,44].

On the other hand, Figure 4b demonstrates the FTIR spectrum of the CS/GL NFs following the incorporation of DAC. The DAC spectrum displays distinct peaks at 3390 cm^−1^, 2958 cm^−1^, and 1720 cm^−1^, which correspond to the abundance of N−H stretching, alkyl C−H stretching, and aromatic C=C bending, respectively. This is in good agreement with what has been previously reported in the literature [45]. Finally, the DAC-CS/GL NFs spectrum displayed in Figure 4b demonstrates the successful incorporation of DAC within the CS/GL NF matrices through revealing the characteristic peaks of DAC within the nanofibers spectrum.

#### 3.3.3. Physicochemical Characterization

In order to investigate the physicochemical features of both the CS/GL NFs and ZnO/DAC-CS/GL NFs, several factors were assessed, which were as follows: (a) in vitro biodegradability, (b) swellability, (c) water vapor permeability, and (d) porosity.

**Figure 5:**
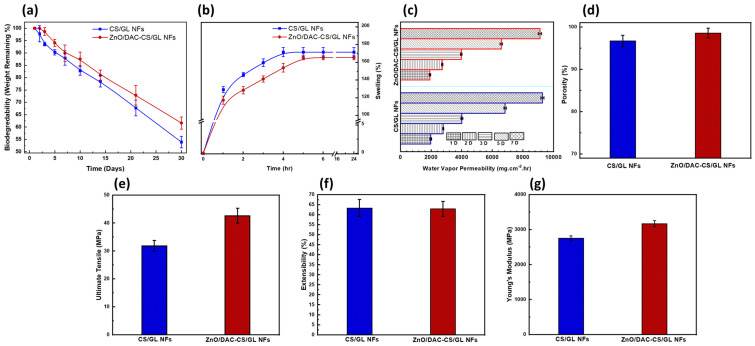
Physicochemical and mechanical characterization of both the CS/GL NFs and ZnO/DAC-CS/GL NFs in terms of the following: (**a**) biodegradability, (**b**) swellability, (**c**) water vapor permeability, (**d**) porosity, (**e**) ultimate tensile, (**f**) extensibility, and (**g**) Young’s modulus, emphasizing the influence of loading the nanofiber matrices with DAC and ZnO NPs. On the other hand, it has been observed that the loaded nanofiber matrices showed a significant increase in the ultimate tensile. This returns to the reinforcement of the developed nanofiber matrices with ZnO NPs.

A biodegradability study was conducted to investigate the capability of the developed nanofibrous medical textiles in decomposing easily and safely. Figure 5a illustrates the biodegradation profiles of both the unloaded and loaded nanofibrous matrices in PBS (pH 5.5) to mimic the microbial infection environment. The results showed that both nanofibrous matrices preserved more than 50% of their weight over a 30 day study. Additionally, it was observed that the ZnO/DAC-CS/GL NFs displayed a slightly slower degradability rate compared to the CS/GL NFs. This was attributed to the abundance of ZnO NPs, which possess a lower biodegradability rate compared to chitosan and gelatin. This was found to be in good agreement with what has been previously reported in some studies [9,34].

To detect the hydrophilicity–hydrophobicity extent of the developed medical textiles nanofibrous matrices, a swellability study was performed. Figure 5b demonstrates that the swelling percentage (S%) of both the unloaded and loaded nanofibrous matrices reached more than a 150% swelling capacity in 4 h and 5 h, respectively. Moreover, there was no statistically significant difference found in the S% values between F1 and F2. Although the ZnO/DAC-CS/GL NFs exhibited a slightly lower swelling index owing to the presence of ZnO NPs within the nanofiber matrices, they still possess a high swellability, indicating their high hydrophilicity. A high swellability indicates a high hydrophilicity of both developed nanofibrous matrices, which reflects the capability of the developed matrices in being able to act as controlled release reservoirs for incorporated cargoes. This was found to be matched with previously reported studies [9,34].

Biomaterials showing a high water vapor permeability (WVP) are considered to be promising candidates for the fabrication of medical textiles. This is attributed to the high porosity of the developed biomaterials, which is mandatory for releasing the incorporated bioactive compounds or materials. Furthermore, a high water vapor permeability reflects the adequate breathability and capability of the developed material in allowing good aeration and facile breathing, preventing the accumulation of humidity that could lead to the onset of headaches, especially in the case of face masks. The WVP test was carried out in accordance with the ASTM standards and lasted for a period of 7 days, as shown in Figure 5c. The results revealed that both nanofibrous matrices possess a considerably high WVP. This is likely owing to the high hydrophilicity of the two developed matrices. Furthermore, a slight decrease in the WVP was observed in the case of the ZnO/DAC-CS/GL NFs compared to their unloaded counterparts. This returns to the abundance of the ZnO NPs, which may decrease the hydrophilicity of the nanofibrous matrices slightly. This was found to be in accordance with what has been previously reported [9].

Finally, porosity was estimated using a pycnometer, as shown in Figure 5d, which illustrates that both nanofibrous matrices showed a high porosity percentage that exceeded 95% of their volume. Furthermore, it was found that there was no significant difference between both matrices in terms of their porosity, which indicates that the incorporation of both DAC and ZnO NPs did not negatively affect the porosity of the developed nanofibrous matrices. These porosity results confirm the results that were obtained regarding the WVP. 

#### 3.3.4. Mechanical Characterization

The mechanical characterization of both the unloaded and loaded nanofibers was carried out in terms of their ultimate tensile, extensibility percentage, and Young’s modulus, as shown in Figure 5e–g, respectively. This was carried out to investigate whether or not the high porosity generated led to a tremendous decrease in the mechanical features of the developed nanofibrous matrices. It was found that both the unloaded and loaded nanofibrous matrices possessed a high ultimate tensile exceeding 30 MPa. This was found to be in good agreement with the reported mechanical features of the chitosan and gelatin-based nanofibers [46].

Furthermore, it was detected that both the unloaded and loaded nanofibrous matrices possess high extensibility percentages, which confirm their high mechanical strength and robustness to be used as durable medical textiles.

Finally, the Young’s modulus results were found to support the ultimate tensile results, where both the unloaded and loaded nanofibrous matrices show a tensile modulus exceeding 2500 MPa. This was deemed to be quite similar to what has been previously reported for chitosan and gelatin-based matrices [46]. Young’s modulus results confirmed that ZnO NPs reinforcements within the CS/GL NF matrices led to an observed increase in the developed nanofibers’ mechanical features.

### 3.4. In Vitro Daclatasvir Release Profile from the CS/GL NFs

**Figure 6:**
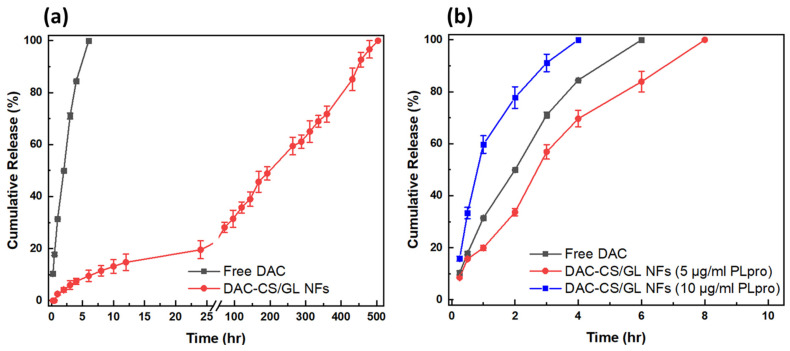
DAC release profile as free DAC and from CS/GL NF matrices in (**a**) PBS medium and (**b**) in the presence of PLpro.

The daclatasvir release profile study was carried out in a PBS buffer medium (pH 5.5) to mimic the actual conditions of microbial infections. Afterward, DAC concentrations at specific time intervals were estimated using a UV–Vis spectrophotometer, following which DAC cumulative release was calculated at specific intervals before being plotted, as shown in Figure 6a. These results demonstrated that free DAC exhibited a complete release within only 6 h. On the other hand, DAC showed a prolonged and sustained release profile from the DAC-CS/GL NF matrices for up to 21 days. These nanofibers released more than 25% of the initially incorporated DAC amount within the first 72 h as a burst release followed by a prolonged and sustained release during the rest of the 21-day study period. The release kinetics of DAC from the DAC-CS/GL NPs were found to follow the Korsmeyer–Peppas model, where the r2 value was detected to be 0.9867. The Korsmeyer–Peppas model is considered a simple model that explains the diffusion of the drug from a polymeric matrix due to water retention within polymeric matrices due to their high swellability. This was found to be in good accordance with the high swellability of the developed matrices, as illustrated in Section 3.3.3.

### 3.5. In Vitro PLpro Activity towards Smart CS/GL NFs

To study the sensitivity of the smart DAC-CS/GL NFs towards PLpro activity, the invasive enzyme secreted by SARS-CoV-2, different concentrations of the enzyme (5 µg/mL and 10 µg/mL, respectively) were added to pre-weighed samples of DAC-CS/GL NFs. This was performed to mimic different SARS-CoV-2 viral loads. Afterward, the amount of released DAC from the nanofibers was estimated at specific time intervals, as shown in Figure 6b. It was found that PLpro enzyme abundance resulted in the rapid release of DAC from the developed nanofibrous matrices. This returns to the PLpro enzyme activity to hydrolyze gelatin within the nanofibrous matrices, leading to DAC release at higher rates compared to the absence of the enzyme. Furthermore, these results proved that increasing PLpro enzyme concentrations led to DAC release at enhanced rates. For instance, adding Plpro at concentrations of 5 µg/mL and 10 µg/mL led to the complete release of DAC from the nanofibers in only 10 h and 4 h, respectively. This confirms that these developed nanofibers can be effective carriers for antiviral drugs, where they can be released rapidly in the case of viral infections. Interestingly, it was also found that DAC can be released completely from the nanofiber matrices at the concentration of only 10 µg/mL of the enzyme.

### 3.6. Antimicrobial Activities of the Unloaded and Loaded CS/GL NFs

**Figure 7:**
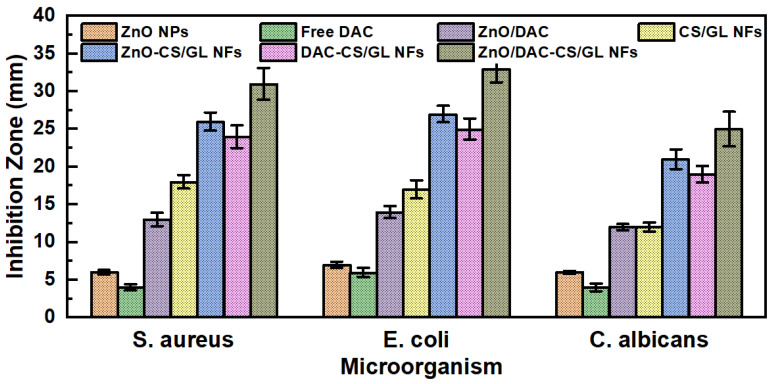
Antimicrobial activity of the individual components of the developed nanofibers, as well as the different unloaded and loaded nanofiber matrices against *S. aureus*, *E. coli*, and *C. albicans*.

ZnO NPs, DAC, ZnO/DAC combination, CS/GL NFs, ZnO-CS/GL NFs, DAC-CS/GL NFs, and ZnO/DAC-CS/GL NFs were all assessed for their antimicrobial activity against *Staphylococcus aureus* (a Gram-positive bacteria), *Escherichia coli* (a Gram-negative bacteria), and *Candida albicans* (a fungus). The antimicrobial assessments of these tested samples were carried out through the inhibition zone technique, where the inhibition zone (mm) for each sample against each pathogen was plotted, as shown in Figure 7.

The results demonstrated that free DAC possessed the lowest antimicrobial activity against the three tested pathogens. It was also observed that ZnO NPs exhibited a higher level of antimicrobial activity against the three tested pathogens compared to that observed with free DAC. However, the combination of both ZnO NPs and free DAC together demonstrated synergistic antimicrobial activity against the tested pathogens, i.e., their antimicrobial activity was higher than the additive individual antimicrobial activity.

Moreover, it was observed that the CS/GL NFs possessed high antimicrobial activity against the three tested pathogens. This returns to the highly reported antimicrobial activity of chitosan owing to its positively charged surface that can interact with and destruct the microorganisms’ walls. This is in good accordance with what has been reported previously [34].

Again, the results demonstrated that both the ZnO-CS/GL NFs and DAC-CS/GL NFs possess synergistic antimicrobial activity against the tested microorganisms strains compared to the same amounts of the individual components.

Finally, the high antimicrobial activity of the ZnO/DAC-CS/GL NFs was proven through the antimicrobial study where they exhibited the highest antimicrobial activity among the tested samples. This returns to the bioactivity of ZnO NPs, DAC, and chitosan.

Hence, ZnO/DAC-CS/GL NF matrices can be considered a promising candidate for the fabrication of bioactive medical textile matrices.

### 3.7. In Vitro Cell Viability and Biocompatibility of ZnO/DAC-CS/GL NFs

**Figure 8:**
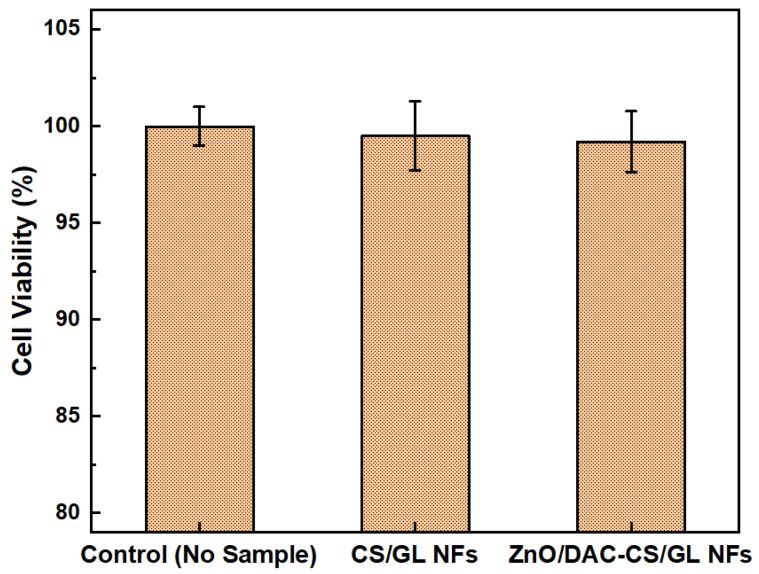
Cell viability of both CS/GL NFs and ZnO/DAC-CS/GL NFs using fibroblasts.

For the cell viability experiment, both CS/GL NFs and ZnO/DAC-CS/GL NFs were selected to evaluate their cell viability and biocompatibility as medical textile matrices, as shown in Figure 8. This was carried out through an MTT assay using a fibroblast cell line.

Cell viability was assessed to confirm that the fabricated nanofiber matrices are safe and biocompatible with the human body as medical textiles. As shown above, both of the tested nanofiber matrices were found to be non-toxic when compared to the negative control (fibroblasts only). Furthermore, there was no significant difference in cell viability found between the CS/GL NF and ZnO/DAC-CS/GL NF matrices, which confirms the biocompatibility of the incorporated ZnO NPs and DAC. Interestingly, the results also showed that the tested nanofibrous matrices exhibited an increase in the optical density of fibroblasts compared to the negative control, where no material was used. This suggests that the developed nanofibrous matrices not only preserved the cell viability, but also provoked cell proliferation, which could lead to the regeneration of damaged skin cells if used in the wound or burn dressing applications.

## 4. Conclusions

This study involved several stages to develop bioactive ZnO/DAC-CS/GL NF matrices to be used as smart medical textiles for protection against microbial and viral infections, including SARS-CoV-2.

During the first stage, daclatasvir was evaluated for its anti-SARS-CoV-2 activity through in silico studies compared to remdesivir and favipiravir, two FDA-approved anti-SARS-CoV-2 agents. Daclatasvir proved its high potential as an inhibitory agent for Mpro and PLpro, two invasive enzymes secreted by SARS-CoV-2. This was confirmed through assessing the Gibbs energy between these three drugs each individually with each of the two enzymes. Daclatasvir showed the highest inhibitory activity against both enzymes. Moreover, it showed higher level of inhibition with PLpro compared to Mpro, which suggests its SARS-CoV-2 inhibitory activity through the PLpro enzyme.

In the second stage, ZnO NPs were successfully prepared through the sol-gel method, a wet chemical technique, before being fully characterized. Morphological investigation using TEM revealed the hexagonal or semi-spherical shape of the ZnO NPs possessing a size of around 15–20 nm. This was also confirmed through XRD, which detected the hexagonal shape of the ZnO NPs and calculated a size of 17.38 using Scherer’s equation. In addition, the chemical composition of these ZnO NPs was confirmed through detecting their characteristic bands using FTIR spectroscopy. Moreover, the formation of ZnO at the nanoscale was further confirmed using UV–Vis spectroscopy, where its excitonic peak was detected at 365 nm and its band gap was calculated to be 3.28 eV using Max Planck’s equation. Finally, DLS measurements confirmed the high stability of the prepared ZnO NPs owing to their detected high positively charged surfaces of 53.42 ± 3.88 mV.

In the third stage, chitosan/gelatin nanofibers (CS/GL NFs) were selected owing to the wide antimicrobial activity of chitosan and the sensitivity of gelatin towards the PLpro enzyme. CS/GL NF matrices were selected to be the smart medical textile base that can incorporate anti-SARS-CoV-2 agents so that they can be degraded as a response to the abundance of PLpro secreted by SARS-CoV-2, resulting in the release of the incorporated anti-viral agents as a response of viral infection. Unloaded and loaded nanofibrous matrices were prepared through the electrospinning technique and were then chemically cross-linked to increase their water stability. Afterward, they were fully characterized to confirm their successful fabrication. For instance, SEM imaging revealed the formation of uniform, bead-free, nanofibrous matrices with a diameter of around 100 nm. It is worth mentioning that the incorporation of ZnO NPs led to the stretching of the produced nanofibers, resulting in a narrower diameter and PDI. Furthermore, TEM imaging revealed the successful incorporation of ZnO NPs within the nanofibrous matrices. Chemical characterization using FTIR confirmed the successful complexation between the chitosan and gelatin chains within the nanofibrous matrices. In addition, DAC incorporation was also confirmed. Physicochemical characterization revealed the high durability of the fabricated nanofibers where they only lost less than 50% of their initial weight over a 30-day study in PBS medium. However, they also revealed their safe and easy biodegradation ability. In addition, swellability showed that the developed nanofibrous matrices showed maximum swellability exceeding 150% of their volume within a few hours. The WVP and porosity results, along with the swellability results, showed that the developed nanofibers are highly porous that can release DAC in a controlled manner. Porosity results also showed that more than 96% of the nanofibers volume is in porous hierarchy. Physicochemical characterization showed that the incorporation of DAC and ZnO NPs within the nanofibrous matrices did not negatively influence their features. Mechanical testing showed that the developed nanofibers demonstrated a high mechanical strength indicated by the high ultimate tensile exceeding 30 MPa, high extensibility % exceeding 60%, and high Young’s modulus exceeding 2.5 GPa, respectively. Furthermore, it was found that the incorporation of ZnO NPs and DAC increased the mechanical strength of the developed nanofibers.

The DAC release profile showed a controlled and sustained release from the CS/GL NF matrices for up to 21 days compared to only 6 h in the case of free DAC. Furthermore, an abundance of the PLpro enzyme showed a prompt and complete release of DAC from the developed nanofibers within only 4–8 h, which was highly comparable to the release of free DAC. This suggests the high potential of the developed nanofibers to act as a smart carrier system for anti-SARS-CoV-2 agents.

Moreover, the cell viability test showed the high biocompatibility of both the unloaded and loaded nanofibers, where normal fibroblasts grew normally at a high rate in the presence of the tested materials.

Finally, antimicrobial studies revealed that the developed ZnO/DAC-CS/GL NF matrices are promising candidates for medical textile fabrication, as in addition to their anti-SARS-CoV-2 activity, they exhibited wide antimicrobial activity against *S. aureus* (Gm + ve bacteria), *E. coli* (Gm -ve bacteria) and *C. albicans* (fungi). The high antimicrobial activity was proven to be a result of the synergistic activity between DAC and ZnO NPs, as well as chitosan within the nanofibrous matrices.

In conclusion, ZnO/DAC-CS/GL NF matrices can be a potential basic material for the fabrication of medical textiles that can help in the prevention of microbial and viral infections, especially for patients and healthcare professionals in hospitals who are highly susceptible to severe microbial and viral infections.

## Figures and Tables

**Table 1:** Gibbs free energy between different ligands with either Mpro or PLpro.

Ligand	Mpro	PLpro
Daclatasvir	−8.5	−9.0
Favipiravir	−4.9	−5.7
Remdesivir	−6.9	−7.2

**Table 2:** A summary of the interactions detected between daclatasvir with either Mpro or PLpro showing the involved amino acids, types of interactions of bonds, and distance of bonds.

Enzyme	Amino Acid	Bond or Interaction	Distance
Mpro	LYS (A:5)	Positive–positive	4.92
		Alkyl	4.26, 4.43
	TYR (A:126)	π–alkyl	4.46
	GLN (A:127)	Carbon–hydrogen	3.74
	ARG (A:131)	π–cation	4.16
	LYS (A:137)	Carbon–hydrogen	3.53
		π–alkyl	5.11
	ASP (A:197)	Carbon–hydrogen	3.45
	THR (A:198)	Carbon–hydrogen	3.48
	LEU (A:286)	Alkyl	5.02
	LEU (A:287)	Alkyl	4.91
	ASP (A:289)	π–anion	3.56
		Attractive charge	5.36
	GLU (A:290)	π–anion	3.51
		Carbon–hydrogen	3.52
PLpro	ASP (A:164)	Carbon–hydrogen	3.51
	ARG (A: 166)	Alkyl	4.48
	PRO (A:247)	Alkyl	5.35
	PRO (A:248)	π–sigma	3.96
	GLN (A:250)	Hydrogen bonding	2.10
	GLU (A:263)	Attractive charge	5.58
	TYR (A:264)	π–sigma	3.78
		π–alkyl	4.01
	TYR (A:268)	Carbon–hydrogen	3.52
		π–alkyl	4.73
	TYR (A:273)	π–alkyl	5.34
	TYR (A:296)	π–alkyl	4.42, 5.15
	PRO (A:299)	π–alkyl	3.72

**Table 3:** Composition of different electrospinning solutions along with their physical properties.

Electrospinning Solution	CS Solution Concentration	GL Solution Concentration	CS: GL Volumetric Ratio	Amount of Incorporated ZnO NPs *	Amount of Loaded DAC ^#^	Viscosity (cP)	Conductivity (mS/cm)
CS/GL	3% *w*/*v* in 1% aqueous acetic acid solution	25% *w*/*v* in 1% aqueous acetic acid solution	1: 1	Zero	Zero	1131 ± 4.97	2.41 ± 0.02
ZnO-CS/GL				10% *w*/*w*	Zero	1673 ± 3.74	2.83 ± 0.03
DAC-CS/GL				Zero	10% *w*/*w*	1495 ± 4.55	2.68 ± 0.04
ZnO/DAC-CS/GL				10% *w*/*w*	10% *w*/*w*	1875 ± 3.94	3.12 ± 0.03

*, ^#^ Amounts of either ZnO NPs or DAC are calculated corresponding to the total amount of polymers in the electrospinning solution.

## Data Availability

All data are available upon request.
